# High-Performance Oxygen Sensing Films with Enhanced Quenching Efficiency and Fluorescence Stability via FITC–PtOEP FRET and Imidazole Modification

**DOI:** 10.3390/ma19122502

**Published:** 2026-06-10

**Authors:** Honglin Zhang, Mingkuan Xu, Ye Phone Myint, Ke Zhang, Caixia Chi, Sai Chen, Tong Zhang, Jiupeng Zhao, Yao Li

**Affiliations:** 1 School of General Education, Guangzhou Huali College, Guangzhou 511325, China; 2 MIIT Key Laboratory of Critical Materials, Technology for New Energy Conversion and Storage, School of Chemistry and Chemical Engineering, Harbin Institute of Technology, Harbin 150001, China; mingkuanxu2021@163.com (M.X.); phonemyintye@gmail.com (Y.P.M.); 21b925054@stu.hit.edu.cn (S.C.); zhangtong010225@163.com (T.Z.); jpzhao@hit.edu.cn (J.Z.); 3 Heilongjiang Province Key Laboratory of Environmental Catalysis and Energy Storage Materials, Food and Pharmaceutical Engineering College, Suihua University, Suihua 152061, China; chicaixia617@163.com; 4 Center for Composite Materials, Harbin Institute of Technology, Harbin 150001, China

**Keywords:** oxygen sensor, photonic crystal, FRET, sensitivity

## Abstract

**Highlights:**

- A FRET system (FITC-PtOEP) was integrated into a photonic crystal film for oxygen sensing.
- Imidazole modification boosted fluorescence intensity by 52.4% under 100% O_2_ atmosphere.
- Imidazole also provided excellent photostability with a 99.77% fluorescence retention rate.

**Abstract:**

Fluorescence resonance energy transfer (FRET) was implemented in a photonic crystal-structured oxygen sensing film by incorporating fluorescein isothiocyanate (FITC) with platinum octaethylporphyrin (PtOEP). The highest Stern-Volmer quenching constant (*K*_SV_ = 20.20 ± 0.25) and maximum quenching ratio (21.81 ± 0.21) were achieved at a PtOEP:FITC molar ratio of 1:1. Compared to the control, the *K*_SV_ and maximum quenching ratio increased by 56.8% and 48.4%, respectively. Additionally, imidazole was introduced into the oxygen-sensing film via a casting method. The results demonstrated that imidazole effectively modulates the energy transfer efficiency between oxygen molecules and PtOEP. Under 20% O_2_ and 100% O_2_ atmospheres, the fluorescence intensity of the imidazole-modified film increased by 18.4% and 52.4%, respectively. Furthermore, imidazole provided excellent protection for PtOEP, yielding a fluorescence retention rate of 99.77% ± 0.18%.

## 1. Introduction

In recent years, optical oxygen sensors have been extensively employed in biosensing, water quality monitoring, aerospace and other fields [1,2,3]. The global oxygen sensor market reached USD 4.24 billion in 2023 and is projected to grow to USD 7.42 billion by 2032. However, their further development is still restricted by inherent bottlenecks, including weak fluorescence/phosphorescence signals, unsatisfactory sensitivity and poor photobleaching resistance. Designing and constructing novel optical oxygen sensors via structural regulation to address the above challenges remains a core research focus in the field of oxygen sensing. A conventional approach is to incorporate high-refractive-index rutile TiO_2_ into oxygen sensing films as light scattering centers [4]. Compared with the simple incorporation of light scattering centers, fluorescence (phosphorescence) resonance energy transfer (FRET/PRET) [5,6] synergistically amplifies fluorescence signals from the perspective of metal-dielectric coupling. Owing to its versatility and designability, this approach has become a mainstream signal enhancement technique in the field of oxygen sensing.

Fluorescence resonance energy transfer (FRET) occurs when the emission spectrum of a donor fluorophore overlaps by more than 30% with the excitation spectrum of an acceptor, and the two are separated by less than 10 nm [7,8]. Under these conditions, the donor can transfer energy non-radiatively to the acceptor, leading to enhanced or modulated fluorescence. Zhao et al. [9] synthesized an amphiphilic graft copolymer (P3) composed of poly(ethylene glycol) methacrylate and polystyrene, and developed oxygen-sensing micelles containing platinum porphyrin (PtTFPP) and a conjugated polymer (CP). Effective FRET between CP and PtTFPP significantly improved the emission intensity and stability of the micelles. Other studies [10,11,12] have explored related FRET mechanisms to enhance the sensitivity and energy-transfer efficiency of porphyrin-based oxygen sensors.

Sensitivity is a critical parameter for evaluating oxygen-sensing films, and many researchers have optimized it by modifying the sensing substrate and probe environment. For instance, fluorinated polymers exhibit excellent thermal and photostability while enhancing oxygen-probe interactions. Amao et al. [13] reported that a fluorinated copolymer doped with an iridium complex exhibited two orders of magnitude higher sensitivity than a non-fluorinated styrene matrix. Mao et al. [11] further improved sensitivity by fabricating hydrophobic microfiber films using PtTFPP-grafted fluorinated polymers, achieving a 584% enhancement due to increased surface area. Similarly, porous substrates have been shown to boost oxygen diffusion and optical response, as reported by Salaris et al. [12] and Mao et al. [13] observed both higher sensitivity and better photostability in porous membranes compared to dense films. The quenching constant of an oxygen sensor depends not only on the probe and host matrix but also on the diffusion and interaction efficiency between oxygen molecules and the fluorophore. By adjusting the film thickness, these interactions and the resulting quenching behavior can be tuned [14]. Zhang et al. [15] demonstrated that thinner PtOEP/poly(p-FSt-co-TFEMA) films exhibited stronger quenching effects and higher signal-to-noise ratios, achieving detection performance comparable to that of commercial Clark electrodes. Fernando at al. [16] developed supramolecular chemistry-based monochromatic and bichromatic fluorescent sensors for sensitive identification of barium ions from neutrinoless double-beta decay, offering a molecular design reference for future particle detection. Samuel at al. [17] constructed an ultrashort-lifetime nano oxygen sensor in biosensing, which eliminates biological autofluorescence, enhances the performance of time-resolved oxygen detection, and promotes oxygen imaging applications in living cells and tissues.

However, the existing PtOEP-based oxygen sensors suffer from weak visible-light absorption, requiring high excitation intensities that accelerate photobleaching, and direct excitation generates singlet oxygen causing cumulative photodamage to both probe and matrix. Reported photonic crystal-enhanced sensors rely solely on slow photon effects or stopband amplification for directly excited probes, lacking integrated signal amplification mechanisms.

In this study, we developed a high-performance oxygen-sensing film based on a photonic crystal metamaterial structure. Platinum octaethylporphyrin (PtOEP) was employed as the oxygen-sensitive probe, while polystyrene (PS) microspheres formed the photonic crystal matrix. By aligning the photonic band gap with the excitation and emission spectra of PtOEP, the system was designed to enhance fluorescence intensity and oxygen sensitivity. Our work establishes a hierarchical architecture wherein the photonic crystal provides the optical scaffold, FRET achieves signal amplification, and imidazole introduces the regulatory layer. FRET-mediated indirect excitation reduces direct PtOEP photoexcitation and mitigates photobleaching, while the orthogonal imidazole layer decouples signal amplification from quenching control, enabling concurrent optimization of sensitivity, and providing deeper insights into performance optimization in photonic crystal-based oxygen sensors.

## 2. Materials and Methods

### 2.1. Materials

PtOEP was obtained from Anolon (Beijing) Biotechnology Co., Ltd. (Beijing, China). Styrene (St) was purchased from Aladdin Reagent (Shanghai) Co., Ltd. (Shanghai, China). Potassium persulfate (K_2_S_2_O_8_), sodium hydroxide (NaOH), and tetrahydrofuran (THF) were supplied by Xilong Chemical Co., Ltd. (Shantou, China). Polydimethylsiloxane ((C_2_H_6_OSi)_n_) was sourced from Dow Corning (Midland, MI, USA). Tween 80 and anhydrous ethanol were provided by Tianjin Fuyu Fine Chemical Co., Ltd. (Tianjin, China) and Tianjin Tianli Chemical Reagent Co., Ltd. (Tianjin, China), respectively. Fluorescein isothiocyanate and imidazole were obtained from Aladdin Reagent (Shanghai) Co., Ltd. (Shanghai, China). High-purity nitrogen (N_2_) and oxygen (O_2_) were supplied by Harbin Tongda Gas Co., Ltd. (Harbin, China), and ultrapure water was prepared in the laboratory.

### 2.2. Preparation of Oxygen Sensing Film

#### 2.2.1. Preparation of PS Microspheres

Polystyrene (PS) microspheres with high monodispersity were synthesized via a soap-free emulsion polymerization method. Since styrene monomer contains a small amount of polymerization inhibitor to prevent self-polymerization during storage and transportation, it was purified by multiple extractions with a 10 wt% NaOH solution and ultrapure water prior to polymerization. The purified styrene monomer was stored under refrigeration for subsequent use.

The polymerization setup consisted of a three-necked flask equipped with a condenser, mechanical stirrer, and oil bath. 150 mL of ultrapure water was added to the three-necked flask, and the oil bath temperature was set to 70 °C. After reaching the target temperature, the stirring speed was set to 350 rpm and nitrogen gas was introduced. The system was saturated with nitrogen under continuous stirring for 15 min. A predetermined amount of styrene monomer was then added. After 15 min, 15 mL of a 1% by mass potassium persulfate solution was added as an initiator. The protective gas was turned off when the solution in the flask turned light blue. After reacting for 10 h, a milky white PS microsphere solution was obtained, which was centrifuged and washed several times and placed in a refrigerator for later use.

#### 2.2.2. Preparation of PS Fluorescent Microspheres

A predetermined amount of PtOEP was dissolved in THF to prepare a 1 × 10^−3^ mol/L PtOEP/THF solution. A measured volume of this solution was added to the PS microsphere emulsion under THF:H_2_O = 1:500 (*v*/*v*). The mixture was ultrasonicated for 10 min and then kept in a dark room for 2 h to allow PtOEP to swell into the PS microspheres, forming PS fluorescent microspheres. The emulsion was ultrasonicated again before use.

#### 2.2.3. Preparation of PS Fluorescent Microsphere Photonic Crystal Film

A quartz plate (1 mm × 13 mm × 30 mm) was placed in a beaker and cleaned with detergent, ultrapure water, alcohol, and ultrapure water in an ultrasonic vibration machine to remove possible impurities on the surface. Grasp one end of the quartz plate with tweezers and use a blow gun to remove any liquid from the surface. Place the plate in a plasma treatment system and treat under vacuum for 10 min to hydrophilize one side of the plate.

The PS fluorescent microsphere emulsion from Section 2.2.2 was transferred to a volumetric flask. The quartz plate was placed with the hydrophilic side facing upward in the flask, which was then stored in a sealed incubator at constant temperature and humidity for 24 h to allow uniform growth of PS microspheres on the surface.

#### 2.2.4. Preparation of PtOEP@PS-PC/PDMS Oxygen Sensing Film

1.0 g of Dow Corning Sylard 184 silicone rubber (polydimethylsiloxane, PDMS) (Dow Inc., Midland, MI, USA) Solution A and 0.1 g of Solution B were weighed into a beaker. The mixture was stirred with a glass rod for 5 min to ensure thorough mixing. The beaker was covered with plastic wrap to prevent dust contamination and left to stand for 20–30 min to remove bubbles generated during stirring. PDMS was added dropwise onto the surface of the photonic crystal, and the gaps in the photonic crystal were filled with PDMS via a casting method. The quartz plate was placed horizontally in a Petri dish and transferred to a constant-temperature oven at 60 °C for 10 h to prepare the photonic crystal sensing film. The specific preparation condition is shown in Table 1:

At a PS microsphere concentration of 0.2 wt%, a certain amount of PtOEP/THF solution was added to maintain a THF:H_2_O ratio of 1:500. The particle sizes of the PS microspheres in the emulsion were adjusted to 167 nm, 225 nm, 241 nm, 277 nm, and 297 nm. All solutions were used to prepare oxygen-sensing films following the same method described above. Meanwhile, an equal volume of PtOEP/THF solution was uniformly coated onto a blank quartz wafer using a tape casting method. This wafer was then encapsulated with PDMS, and the resulting oxygen-sensing film served as the control group.

#### 2.2.5. Preparation of PtOEP/FITC@PS-PC Oxygen Sensing Film

**Figure 1:**
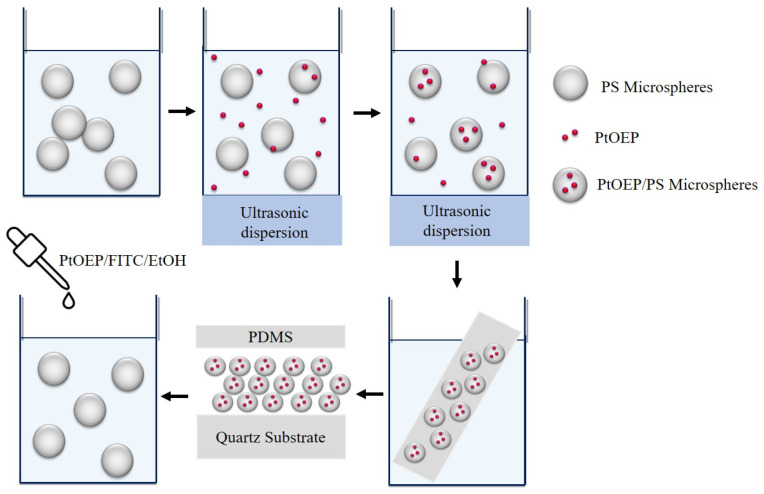
Schematic diagram for fabrication of PtOEP/FITC@PS-PC oxygen sensing film.

Fluorescein isothiocyanate (FITC) was dissolved in ethanol to a specific concentration. A specific amount of FITC/EtOH solution was added to the PS microsphere emulsion described above. The amount of FITC/EtOH added was controlled to achieve a PtOEP:FITC molar ratio of 20:1, 10:1, 1:1, and 1:10 in the PS microsphere solution. A PS microsphere emulsion without FITC was used as the control. All these PS microsphere emulsions were placed in a constant temperature and humidity incubator for slow assembly to form FRET-based oxygen-sensing films, and the schematic diagram of the whole preparation process is presented in Figure 1.

#### 2.2.6. Preparation of PtOEP@PS-PC/Imidazole Oxygen Sensing Film

0.25 g of imidazole crystals was dissolved in 5 mL of anhydrous ethanol to prepare an imidazole solution with a concentration of 0.05 g/mL. A pipette with a range of 5–50 μL was used to measure 0 μL, 10 μL, 20 μL, 30 μL, and 40 μL of the imidazole/ethanol solution, which was then slowly added to the photonic crystal oxygen-sensing film via a casting method. After the ethanol on the film had completely evaporated, oxygen-sensing films with different imidazole addition amounts were obtained.

### 2.3. Instruments and Characterization

The particle size distribution and zeta potential of the samples were measured using a nanoparticle size potentiometer (Nano-ZS90, Malvern Instruments Ltd., Malvern, UK). High-speed separation was performed using a refrigerated centrifuge (GL-21M, Hunan Xiangyi Laboratory Instrument Development Co., Ltd., Changsha, China). UV–Vis absorption measurements were carried out using a spectrophotometer (Lambda 45, PerkinElmer, Shelton, CT, USA). Sample mixing and dispersion were performed using a digital constant-speed electric stirrer (NY-20LS, Enyi Instruments, Huizhou, China) and an ultrasonic cleaner (KQ100E, Kunshan Ultrasonic Instruments Co., Ltd., Kunshan, China). Fluorescence spectra were obtained using an LS-55 fluorescence spectrophotometer (PerkinElmer, Waltham, MA, USA). Under the environmental conditions of 45% relative humidity (R.H.) and 25 °C, the relevant parameters of the spectrometer are set as follows: the slit width is set to 10 nm, the scanning speed is 500 nm/min, and the equilibration time is 3 min. The gas flow control was achieved using a mass flow controller (100 MS, Beijing Porter Instruments, Beijing, China) and the gas flow rate during the measurement is 0.5 L/min. Fiber-optic detection was carried out using a Maya 2000 Pro spectrometer (Marine Optics, Dana Point, CA, USA) together with a 10 mm quartz cuvette (Yixing Spectro-Optics Optical Components Factory, Yixing, China). The surface morphology and microstructure of the samples were characterized using a field-emission scanning electron microscope (SUPRA 55 SAPPHIRE, Carl Zeiss, Oberkochen, Germany).

## 3. Results and Discussion

### 3.1. Optical Characterization and FRET-Based Enhancement of PtOEP/PS Photonic Crystal Oxygen Sensing Films

**Figure 2:**
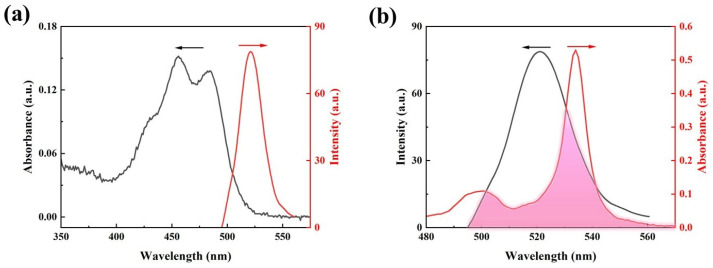
(**a**) FITC absorption spectrum and fluorescence spectrum; (**b**) Intersection of PtOEP absorption spectrum and FITC fluorescence spectrum.

A certain amount of FITC was dissolved in anhydrous ethanol to prepare a FITC/EtOH solution with a concentration of 1 × 10^−3^ mol/L. 3 mL of this solution was placed in a quartz cuvette, and the absorption peak of FITC was measured using a UV-visible spectrophotometer. The results showed that FITC exhibited strong absorption between 400 and 500 nm and a maximum emission peak at 520 nm, as shown in Figure 2a. Plotting the FITC emission peak against the Q-band absorption peak of PtOEP (Figure 2b) revealed a significant overlap, which provides the necessary condition for FRET. In this system, FITC acts as the donor and PtOEP as the acceptor.

### 3.2. Microstructural Characterization of PtOEP/FITC@PS Photonic Crystal Oxygen Sensing Film

**Figure 3:**
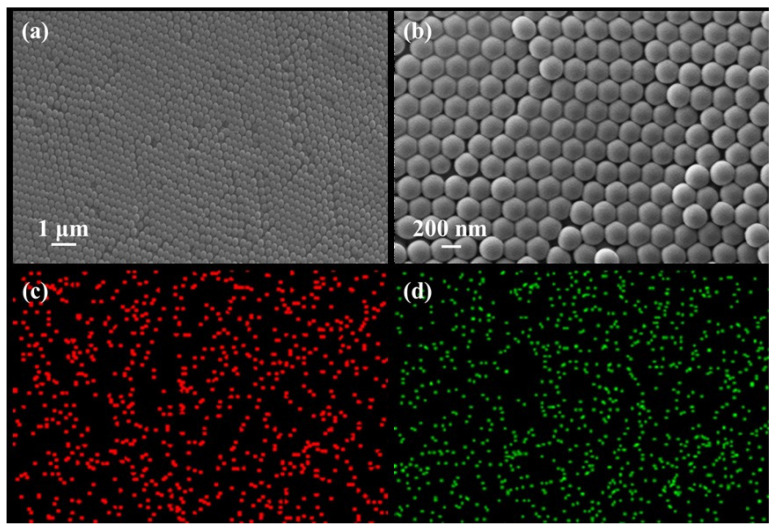
PtOEP/FITC@PS photonic crystal structure: (**a**) 20,000×; (**b**) 80,000×; (**c**) EDS image of the photonic crystal—Pt elemental scan; (**d**) EDS image of the photonic crystal—S elemental scan.

Figure 3 shows a photonic crystal prepared by co-swelling PtOEP and FITC into PS microspheres. Figure 3a presents a surface image of the photonic crystal structure at 20,000× magnification, revealing the presence of some point and surface defects. These defects arise because the photonic crystal was grown in a constant temperature and humidity incubator (non-ideal conditions). Figure 3b shows a cross-sectional SEM image (The acceleration voltage was set at 30 kV) of the photonic crystal at 80,000× magnification, indicating that the PS microspheres are arranged in a highly regular face-centered cubic (fcc) structure. No significant differences were observed between the photonic crystals prepared with and without FITC (Figure 3a,b), demonstrating that the introduction of FITC does not affect photonic crystal growth. Figure 3c and Figure 3d show the distribution of the Pt element (from PtOEP) and S element (from FITC) in the photonic crystal, respectively. Both elements are uniformly distributed without obvious aggregation, indicating that the swelling method effectively embeds PtOEP and FITC into the interior of the PS microspheres, thereby achieving good dispersion.

### 3.3. Oxygen Sensing Film Sensing Performance Test

The oxygen-sensing film containing PtOEP/FITC was tested using an LS-55 fluorescence spectrophotometer (PerkinElmer Inc., Waltham, MA, USA) with an excitation wavelength of 380 nm, and the performance of the oxygen sensing film under different molar ratios was explored.

**Figure 4:**
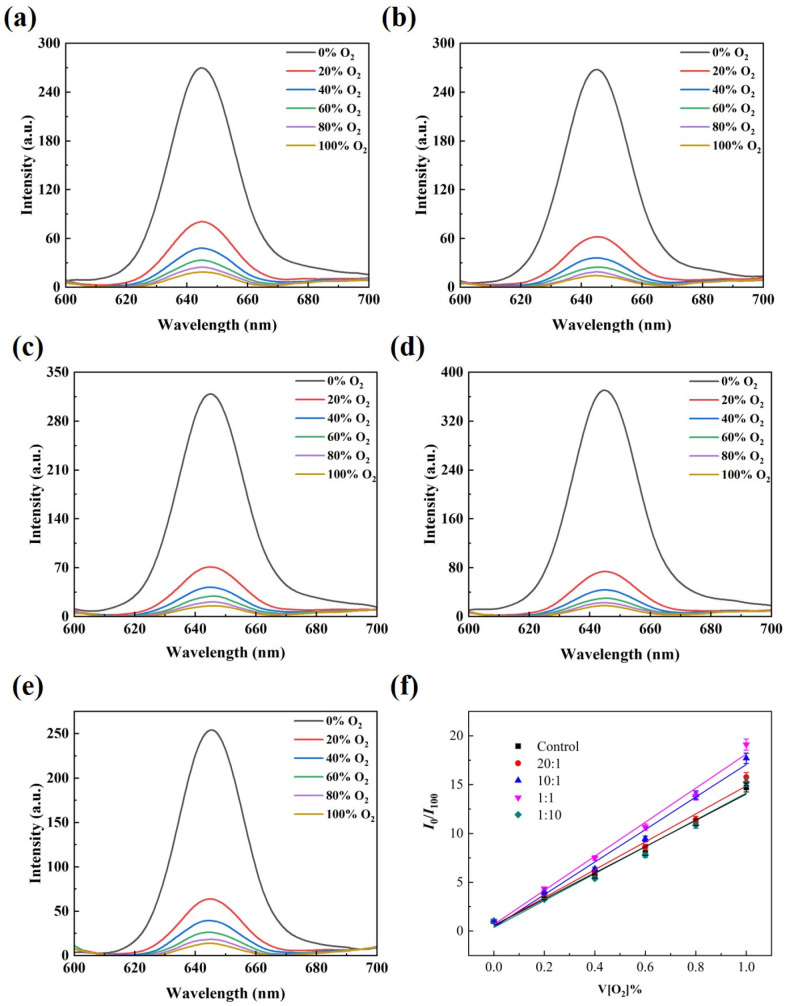
Fluorescence Response Curves: (**a**) Control; (**b**) 20:1; (**c**) 10:1; (**d**) 1:1; (**e**) 1:10; (**f**) Linear S-V Curves.

FITC was dissolved in ethanol, and a small amount of FITC/EtOH solution was added to the PS microsphere emulsion containing a fixed amount of PtOEP. The amount of FITC/EtOH added was adjusted so that the molar ratio of PtOEP to FITC was 20:1, 10:1, 1:1, and 1:10, respectively. The PS microsphere emulsion without FITC was used as the control group. The oxygen content in the quartz cuvette was increased from 0% to 100% in increments of 20%, and the fluorescence intensity at 645 nm was fitted to the Stern-Volmer equation [18]. Figure 4 shows that with increasing FITC content, the fluorescence intensity of the oxygen sensing film initially increases and then decreases.

At a molar ratio of 1:1, the fluorescence intensity of PtOEP increases by 37.5% compared to the control. This phenomenon is consistent with a FRET mechanism arising from the short intermolecular distance between PtOEP and FITC molecules within polystyrene (PS) microspheres, thereby resulting in a significant enhancement of the PtOEP fluorescence signal. As the number of FITC molecules in the PS microspheres increases, the intermolecular distance decreases, leading to aggregation and “self-quenching”. This reduces the energy supplied by FITC to PtOEP, resulting in a decrease in the fluorescence intensity of the oxygen sensing film. The oxygen-sensing film with a PtOEP:FITC molar ratio of 1:1 exhibited optimal sensitivity, with a quenching constant of 20.20 ± 0.25. All oxygen-sensing films had R^2^ values above 0.99, indicating that the FRET-based oxygen-sensing films have high relative measurement accuracy.

**Figure 5:**
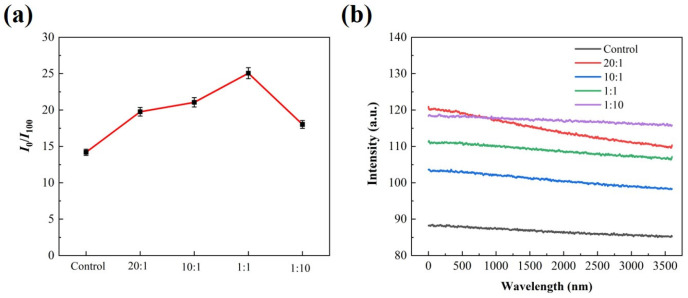
(**a**) Relationship between maximum quenching ratio and molar ratio; (**b**) Photostability curves at different molar ratios.

The maximum quenching ratio of the PtOEP/FITC photonic crystal sensor film was measured at room temperature. Figure 5a shows that as the FITC content in the PS microspheres increased, the fluorescence intensity of the oxygen-sensing film under nitrogen saturation first increased and then decreased. At a PtOEP:FITC molar ratio of 1:1, the fluorescence intensity was 37.5% higher than that of the control group. The quenching constant (20.20 ± 0.25) and maximum quenching ratio (21.81 ± 0.21) of the oxygen-sensing film also reached their maximum values. This is due to energy transfer between FITC (donor) and PtOEP (acceptor): the energy emitted by FITC is reabsorbed by PtOEP, increasing the fluorescence intensity of PtOEP. Thus, increasing FITC content initially increases the fluorescence intensity of PtOEP. However, when the FITC content exceeds a certain threshold, the intermolecular distance between FITC molecules decreases, causing aggregation and self-quenching [19,20]. As a result, the energy supplied by FITC to PtOEP decreases, leading to a reduction in the fluorescence intensity of the oxygen-sensing film. Meanwhile, under oxygen-saturated conditions, the fluorescence intensity of PtOEP does not change significantly, causing the maximum quenching ratio of the oxygen-sensing film to also exhibit a trend of first increasing and then decreasing.

The oxygen-sensing films prepared under the control condition and different PtOEP:FITC molar ratios were continuously irradiated with 380 nm excitation light for 1 h using a fluorescence spectrometer, and the change in fluorescence intensity was measured. Figure 5b shows the photostability curves of the oxygen-sensing films, which correspond to the control group and PtOEP:FITC molar ratios of 20:1, 10:1, 1:1, and 1:10, respectively. All oxygen-sensing films exhibited a photostability of 90%, indicating that the addition of FITC had no effect on the photodegradation rate of PtOEP. No obvious regular changes were observed in the photostability of the oxygen-sensing films with FITC, confirming that FITC does not significantly affect the photostability of PtOEP.

### 3.4. Study of the Effect of Imidazole on the Sensing Performance of Photonic Crystal Oxygen Sensing Films

#### 3.4.1. Microstructural Characterization of PtOEP@PS-PC/Imidazole Oxygen Sensing Film

**Figure 6:**
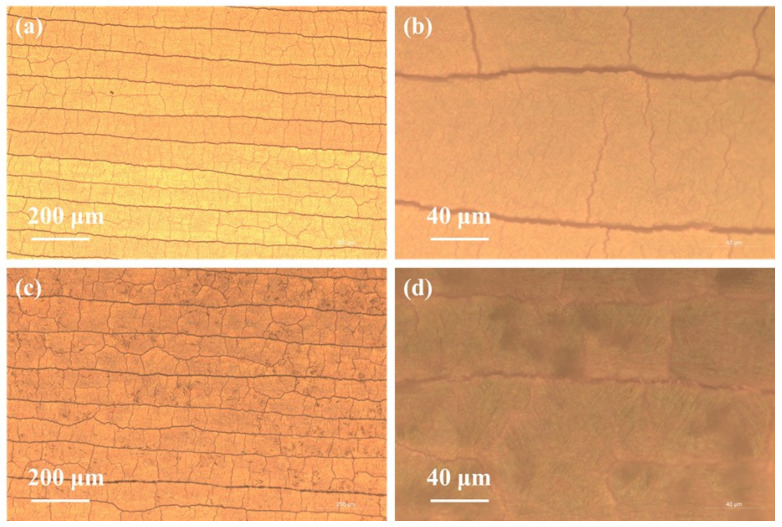
Microscope images of the photonic crystal oxygen sensing film: (**a**) Surface of the sensing film under a 10× objective lens; (**b**) Surface of the sensing film under a 50× objective lens; (**c**) Surface of the imidazole/oxygen sensing film under a 10× objective lens; (**d**) Surface of the imidazole/oxygen sensing film under a 50× objective lens.

Figure 6 shows the surface changes in the oxygen-sensing film (12.12 ± 0.11 μm) after adding 20 μL of a 0.05 g/mL imidazole/ethanol solution. Figure 6a,b show the oxygen-sensing film without imidazole, with a clean surface free of minor impurities. Figure 6c,d show the oxygen-sensing film after imidazole addition. Figure 6c reveals several areas of black material; increasing the objective magnification from 10× to 50× (Figure 6d) shows that this is due to the evaporation of ethanol from the imidazole/ethanol solution, leaving imidazole residues on the film surface. Significant changes in the optical characteristics of the sensing film were also observed.

#### 3.4.2. Fluorescence Performance Test Under Different Gas Atmospheres

In a 20% O_2_ atmosphere, the fluorescence intensity of the oxygen-sensing film first increased and then decreased with the addition of the imidazole/ethanol solution. Taking the fluorescence intensity of the oxygen-sensing film without imidazole in a 20% O_2_ atmosphere as the control, the enhancement factors were 1.046, 1.171, 1.184, and 1.070 for imidazole/ethanol addition amounts of 10 μL, 20 μL, 30 μL, and 40 μL, respectively. The highest fluorescence intensity and optimal signal to noise ratio were achieved when 30 μL of the solution was added. This is because imidazole provides a certain degree of protection for platinum porphyrin [21]; the presence of imidazole reduces the quenching effect of oxygen on platinum porphyrin, leading to a gradual increase in fluorescence intensity in the presence of oxygen. With further increases in imidazole addition, the imidazole solution gradually accumulates on the photonic crystal film as ethanol evaporates. When the imidazole concentration reaches a certain level, imidazole crystals affect the optical properties of the film, resulting in a decrease in fluorescence intensity.

**Figure 7:**
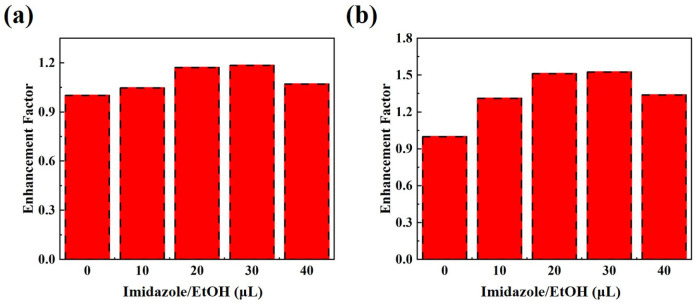
Fluorescence performance test of oxygen sensing film under different gas atmospheres: (**a**) Change in enhancement factor with the amount of imidazole/ethanol solution added under 20% O_2_ atmosphere); (**b**) Change in fluorescence enhancement with the amount of imidazole/ethanol solution added under 100% O_2_ atmosphere.

As shown in Figure 7b, under oxygen-saturated conditions (100% O_2_), the fluorescence intensity also first increased and then decreased with the addition of the imidazole/ethanol solution. Taking the fluorescence intensity of the oxygen-sensing film without imidazole under oxygen saturation as the control, the enhancement factors were 1.311, 1.511, 1.524, and 1.339 for imidazole/ethanol addition amounts of 10 μL, 20 μL, 30 μL, and 40 μL, respectively. A significant increase in the enhancement factor was observed under oxygen saturation compared with that under 20% O_2_, which is attributed to the higher oxygen content. For the oxygen-sensing film, imidazole inhibits the quenching effect of oxygen molecules on platinum porphyrin molecules; in contrast, for the film without imidazole, the quenching effect of oxygen molecules on the probe is enhanced, leading to differences in the enhancement factor.

#### 3.4.3. Establishment of Stern-Volmer Linear Equation

**Figure 8:**
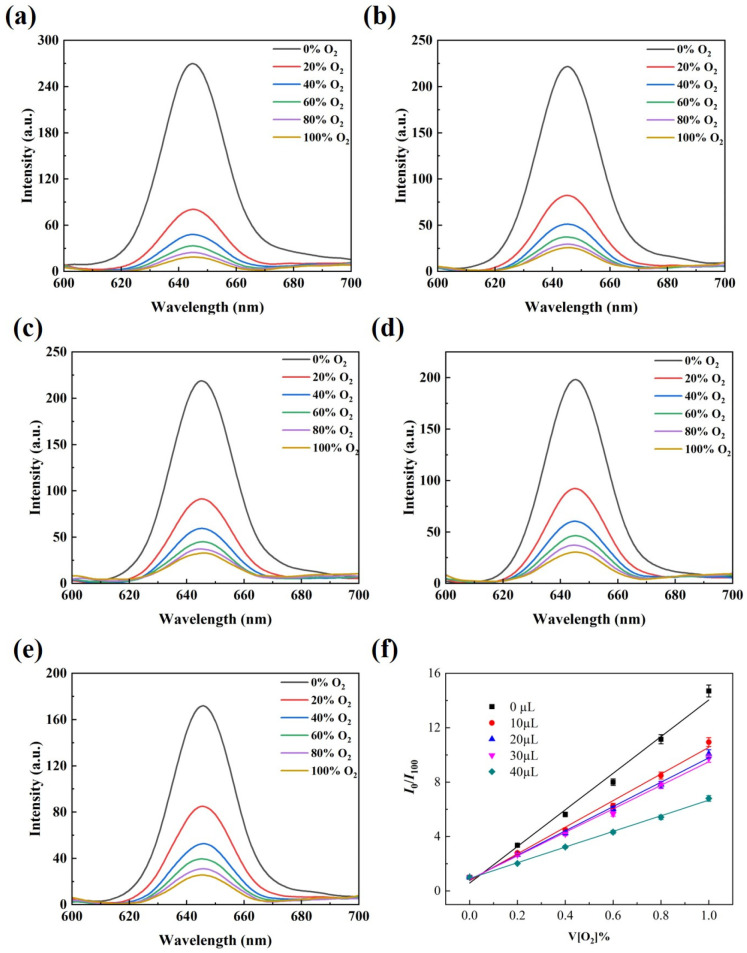
Fluorescence Response Curves: (**a**) 0 μL; (**b**) 10 μL; (**c**) 20 μL; (**d**) 30 μL; (**e**) 40 μL; (**f**) Linear S-V curves.

Figure 8 shows the fluorescence spectra of the oxygen-sensing film with different imidazole/ethanol addition amounts and the corresponding Stern–Volmer equations. Figure 8a–e correspond to imidazole/ethanol addition amounts of 0 μL, 10 μL, 20 μL, 30 μL, and 40 μL, respectively. All spectra show that as the oxygen content increased from 0% to 100%, the fluorescence intensity decreased significantly, especially for the oxygen-sensing film without imidazole. This is because imidazole regulates the energy transfer efficiency between oxygen molecules and probe molecules and provides protection for the probe, resulting in a slower decrease in fluorescence intensity for the imidazole-modified films. For all spectral data, 645 nm remained the maximum emission wavelength of the oxygen-sensitive probe, indicating that the addition of imidazole did not cause a shift in the emission peak.

Under nitrogen saturation, the fluorescence intensity gradually decreased with increasing imidazole content. This is because in the absence of oxygen, imidazole has no effect on regulating energy transfer efficiency. The presence of imidazole crystals causes slight scattering of the fluorescence emission, thereby reducing the fluorescence intensity [19]. Under oxygen saturation, the fluorescence intensity first increased and then decreased with increasing imidazole content. This is due to the protective effect of imidazole on the oxygen-sensitive probe. However, excessive imidazole causes significant optical scattering of the fluorescence intensity.

The linear Stern-Volmer equation data based on fluorescence intensity showed that the sensitivity of the oxygen-sensing film first decreased and then increased with increasing imidazole content, reaching the lowest value when 30 μL of the imidazole/ethanol solution was added. When 30 μL of the imidazole/ethanol solution was added, the fluorescence intensity under oxygen saturation was 52.4% higher than that of the oxygen-sensing film without imidazole, indicating effective regulation of the energy transfer efficiency between oxygen molecules and the oxygen-sensitive probe. Additionally, all oxygen-sensing films exhibited Stern-Volmer linear relationships with R^2^ values above 0.99; the maximum R^2^ value (0.9997) was achieved when 30 μL of the imidazole/ethanol solution was added, indicating high relative detection accuracy. Therefore, considering the accuracy and stability of oxygen content testing as well as the signal to noise ratio during the measurement process, the optimal addition amount of imidazole is determined to be 30 μL.

#### 3.4.4. Cyclic Stability Test

**Figure 9:**
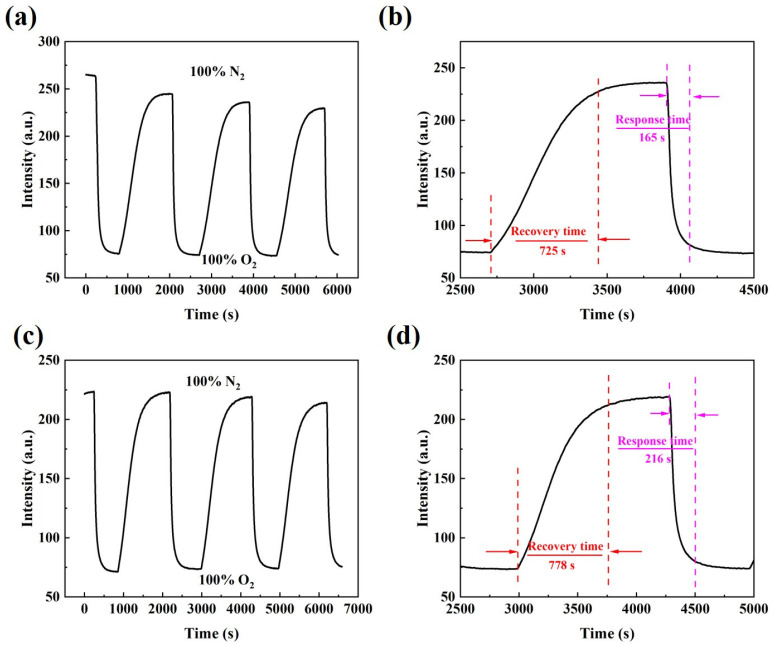
Cyclic Stability Test of Oxygen Sensing Film: Imidazole/Ethanol 0 μL Oxygen Sensing Film: (**a**) Cyclic Stability Curve; (**b**) Response Time Calculation Curve Imidazole/Ethanol 30 μL Oxygen Sensing Film: (**c**) Cyclic Stability Curve; (**d**) Response Time Calculation Curve.

The response time and recovery time of the oxygen sensor film in different gas atmospheres are defined as the time it takes for the fluorescence intensity to decay and recover to 95%, respectively [22]. Figure 9 shows the cyclic stability and response time curves of the oxygen sensor film when no imidazole was added and when the imidazole/ethanol solution was added at a concentration of 30 μL.

Figure 9a and Figure 9c show the dynamic response curves of the oxygen-sensing film without imidazole and the film with 30 μL of the imidazole/ethanol solution after three cycles of nitrogen saturation and oxygen saturation, respectively. After three cycles, the fluorescence intensity of the film without imidazole decreased by 12–13%, while that of the imidazole-modified film decreased by only 4–5%. These results indicate that the oxygen-sensing film without imidazole has poor cyclic stability and reproducibility, whereas the imidazole-modified film exhibits good cyclic reversibility. This suggests that imidazole improves the cyclic stability of the oxygen-sensing film and reduces the photodegradation of the oxygen-sensitive probe under continuous excitation.

From Figure 9b,d, the response time of the oxygen-sensing film without imidazole was 165 s and the recovery time was 725 s. In contrast, the response time and recovery time of the imidazole-modified film were 216 s and 778 s, respectively. Meanwhile, all recovery times were longer than the response times, which is attributed to the strong attraction between the oxygen-sensitive probe molecules and oxygen, requiring more time for the film to switch from an oxygen-rich environment to a nitrogen-rich environment.

#### 3.4.5. Light Stability Test

**Figure 10:**
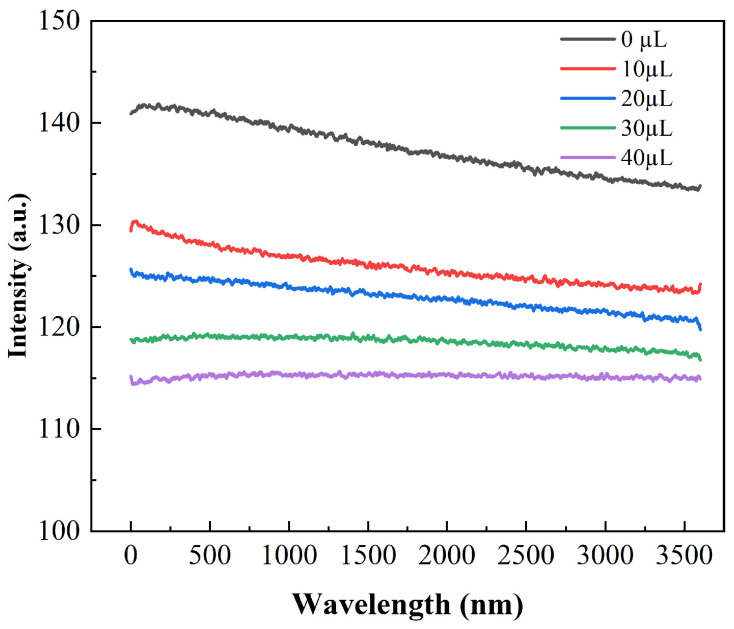
Photostability of oxygen sensing films at different imidazole/ethanol solution addition levels: (0 µL; 10 µL; 20 µL; 30 µL; 40 µL).

Figure 10 shows the change in fluorescence intensity of oxygen-sensing films with different imidazole/ethanol addition amounts under continuous irradiation with 380 nm excitation light for 1 h, which corresponds to imidazole/ethanol addition amounts of 0 μL, 10 μL, 20 μL, 30 μL, and 40 μL, respectively. With increasing imidazole/ethanol addition, the fluorescence retention rate increased from an initial 95.01% ± 0.32% to a final 99.77% ± 0.18%. This phenomenon occurs because although the swelling of PS microspheres embeds some of the oxygen-sensitive probe “inside” the microspheres, a portion remains adsorbed on the surface. The presence of imidazole further protects the oxygen-sensitive probe, reducing the degradation rate of PtOEP under continuous laser irradiation and improving its resistance to photobleaching. Thus, the fluorescence retention rate generally increased with increasing imidazole/ethanol addition. The fluorescence retention rate of the oxygen-sensing film increased with imidazole addition, ultimately reaching 99.77% ± 0.18%, significantly enhancing the film’s resistance to photobleaching.

Furthermore, among the relevant studies (Table 2), recent reports have demonstrated that the sensitivity of optical oxygen sensors can be significantly enhanced by incorporating PtOEP into polymer matrices. Comparative analysis reveals that the present system exhibits the strongest photostability and relatively high sensitivity among reported counterparts, whereas the prolonged response and recovery times are attributed to the robust host-guest interaction between imidazole and the luminophore. Furthermore, in fields such as long-term environmental monitoring, industrial process control, and soil oxygen profile analysis, oxygen concentrations typically change gradually due to slow diffusion in porous media or large-scale atmospheric mixing. Measurements are usually taken at fixed intervals rather than continuous real time tracking. Therefore, such relatively long response times are more acceptable.

## 4. Conclusions

In this study, we have developed a hierarchically structured oxygen-sensing film based on PS microspheres with a large specific surface area, and systematically elucidated the synergistic mechanisms through which FITC and imidazole modulate the photophysical and sensing performance of the integrated platform. The incorporation of FITC into PS microspheres establishes an efficient FRET pathway with surface-adsorbed PtOEP, wherein the substantial spectral overlap between FITC emission and PtOEP absorption provides the fundamental prerequisite for non-radiative energy transfer. At the optimal PtOEP:FITC molar ratio of 1:1, the fluorescence intensity in a nitrogen atmosphere increased by 37.5%, the Stern-Volmer quenching constant rose from 12.88 ± 0.13 to 20.20 ± 0.25, and the maximum quenching ratio reached 21.81 ± 0.21. Then, the oxygen-sensing performance was regulated by adding imidazole. The addition of 30 μL imidazole/ethanol solution enhanced fluorescence intensity by 18.4% under 20% O_2_ and by 52.4% under 100% O_2_, demonstrating that imidazole provides a tunable control layer for oxygen quenching dynamics. Furthermore, increasing the imidazole content improved the photostability of the films; with 40 μL of the imidazole/ethanol solution, a remarkable fluorescence retention rate of 99.77% ± 0.18% was achieved, highlighting imidazole’s role in enhancing the film’s resistance to photobleaching. Overall, these results show that precise tuning of the FITC and imidazole contents can significantly enhance both the sensitivity and stability of PS-based oxygen-sensing films, providing a promising strategy for the development of high-performance optical sensing applications. In addition, it is expected to construct miniaturized and rapidly responsive oxygen-sensing microdevices by integrating microfluidics or flexible electronics technology, meeting the demand for real-time and intuitive detection of oxygen concentration in dynamic and complex systems.

## Figures and Tables

**Table 1:** Preparation condition of oxygen sensing film.

Sample Number	Microsphere Solution (mL)	Microsphere Concentration (wt%)	THF:H_2_O	Assembly Temperature (°C)	Assembly Time (h)	PDMS Curing Temperature (°C)
1	15	0.05	150	60	24	60
2		0.1				
3		0.2				
4		0.3				
5		0.4				

**Table 2:** Performance comparison of porphyrin-based oxygen sensing films in different polymer matrices.

Material	Response Time (s)	Recovery Time (s)	*K*_SV_	*I*_0_/*I*_100_	R^2^	Photostability (%)
PtOEP/PS [23]	30	200	2.21	3.35	0.9894	97.8
Acrylic substrate; PS/Xylene (PtTFPP/THF) [24]	101	184	18.01	19.12	0.9916	98.1
PEGDA hydrogel (Ru(Ph_2_phen)_3_Cl_2_) [25]	600	750	2.45	3.47	0.9902	98.7
Trifluoroethyl Methacrylate (P(TPP-TFE)) [26]	150	760	\	\	\	94.4
This Work	165	725	20.20	21.81	0.9997	99.7

## Data Availability

The original contributions presented in this study are included in the article. Further inquiries can be directed to the corresponding authors.
